# Supplementary material for: Magnetically controllable multimode interference in topological photonic crystals
Source: Light Sci Appl. 2024 May 14;13:112. doi: 10.1038/s41377-024-01433-1 (PMC11091070; doi:10.1038/s41377-024-01433-1)
Supplement: Supplementary file 1 — Supplemental Information [file 41377_2024_1433_MOESM1_ESM.docx]

***Supplemental Information for*
Magnetically controllable Multimode Interference in topological Photonic Crystals**

Weiyuan Tang^1#^, Mudi Wang^2#^, Shaojie Ma^1^, Che Ting Chan^2*^, Shuang Zhang^1*^

^1^Department of Physics, University of Hong Kong, Hong Kong, China

^2^Department of Physics, The Hong Kong University of Science and Technology, Hong Kong, China

^#^these authors contributed equally to this work

**Supplemental Note 1 Experimental setup**

All YIG rods in the heterostructure waveguide system have a height of 10 mm with a permittivity$\epsilon=13.8\epsilon_{0}$, saturation magnetization 4π𝑀𝑠 = 1850 G and linewidth Δ𝐻 = 50 Oe. When a static magnetic field is applied along the YIG rod, the permeability tensor is given by:

$\mu=\mu_{0}\left( -\begin{matrix} \mu_{r} & i\kappa& 0 \\ i\kappa& \mu_{r} & 0 \\ 0 & 0 & 1 \end{matrix} \right)$ (1)

where $\mu_{r} = 1 +\frac{\omega_{m}\left( \omega_{0}+i\alpha\omega\right)}{\left( \omega_{0}+i\alpha\omega\right)^{2}-\omega^{2}}$ and $\kappa= \frac{\omega_{m}\omega}{\left( \omega_{0}+i\alpha\omega\right)^{2}-\omega^{2}}$. $\omega_{0} = \gamma H_{0i}$ represents the resonance frequency, $\omega_{m} = 4\pi\gamma Ms$ denotes the characteristic frequency and $\gamma= 2.8 \mathrm{MHz}\mathrm{Oe}^{-1}$ is the gyromagnetic ratio. Note that bandgap of our model is far from the resonance frequency of the permeability tensor elements $\mu_{r}$ and $\kappa$, as exemplified in Fig. S1 for $H_{0i}=0.2 T$. Thereby, the weak dispersion in permeability elements within the frequency range of interest is negligible in relevant numerical simulation. Based on the lattice structure of waveguide (Fig. 2a in the main text), two aluminum bottom plates were prepared with 624 holes (larger than the specific rod by 0.2 mm) drilled into the bottom. One of the plates is 12 mm in height and utilized to fix the NdFeB permanent magnet pillars (11 mm height and 4 mm diameter), which provide magnetization for the YIG rods and are embedded into these holes. The other thinner plate (3 mm height) is used to support the YIG rods. Each magnet pillar provides an external magnetic field $H_{0i}$ to the YIG rod on it. The value of $H_{0i}$ acts a function of the distance between the YIG rod and the magnet pillar (Fig. S2), which make it possible to tune the external magnetic field. Finally, the YIG rods are covered by a third aluminum plate to form a 2D waveguide. The right and left boundaries of the sample are clipped by the aluminum plates, which mimic perfect electric conductors. Some circular holes (2 mm in diameter) for excitation are located near the right boundary of the sample, while two slots (2 mm in width), one between domain A and domain B and the other locates near the left boundary, are drilled in the upper plate for detection. The needle source is placed 5 mm below the upper plate through the slot for measurement. The errors of experimental measurement in frequency are less than 1%.

**Supplemental Note 2 Coupling between the chiral edge modes and the width of air gap**

We here explore the correlation between the width of the air gap and the behaviors of the chiral edge modes. The projected band structure of the whole heterostructure waveguide with distinct width of the air gap are illustrated in Fig. S3a. When the width of the air gap is about 0.62a (the interval of two adjacent sites is just equal to the unit cell period $a$), the dispersions of two CEMs are nearly parallel to each other, which is also verified in Fig. S3b with the phase difference of two CEMs being almost constant regardless of varying frequency. With the increase in the width of air gap, the dispersion slope of mode 1 gradually decreases and the phase difference $\Delta\varphi$ shows a stronger dependence on frequency. Specifically, the larger the air gap is, the steeper the curve is, as shown by Fig. S3b.

**Supplementary Figures**


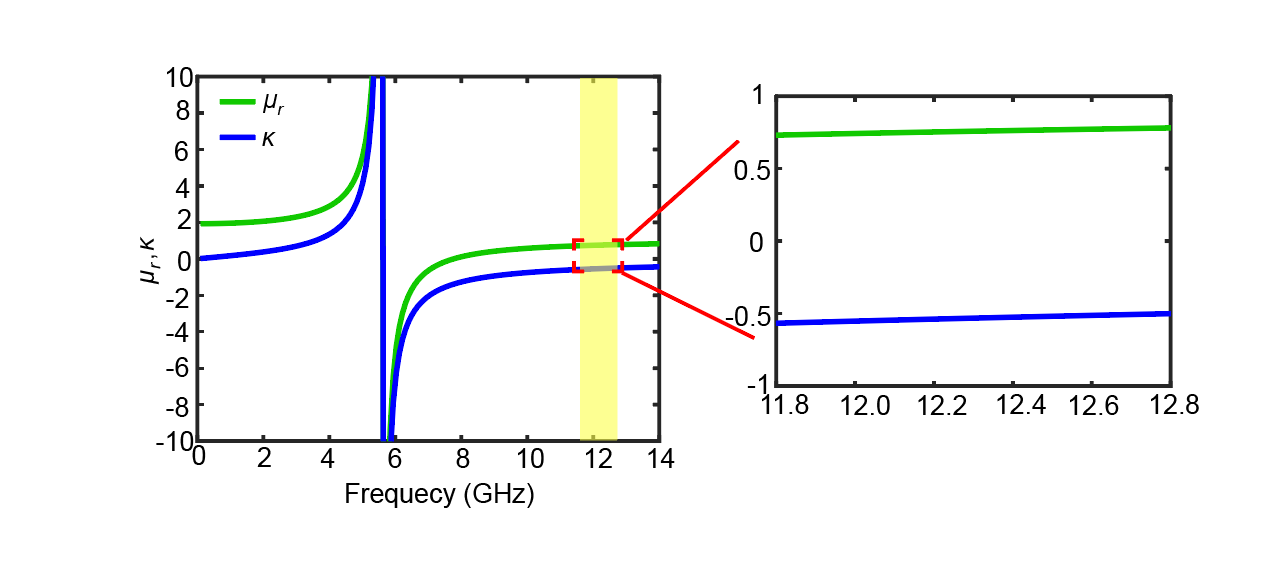


**Fig. S1** **The permeability tensor elements *μ_r_* and *κ* as functions of frequency for *B* = 0.20 T.** The yellow region indicates the bandgap of the photonic crystal.


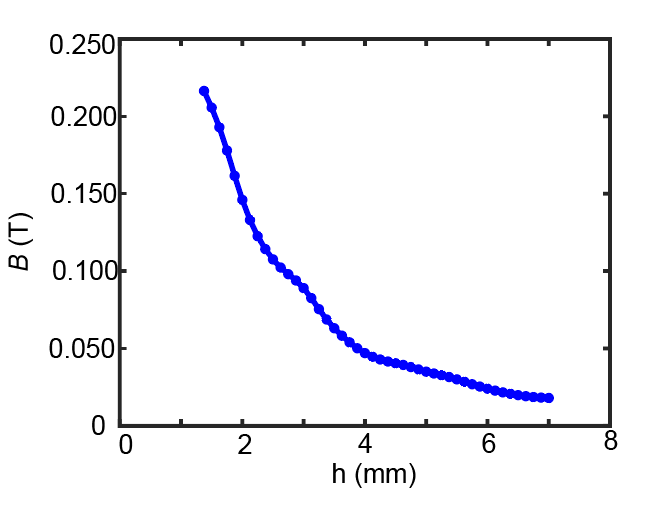


**Fig. S2** **The external magnetic field applied to the YIG rod as a function of the distance** $\boldsymbol{h}$**from the magnet.**


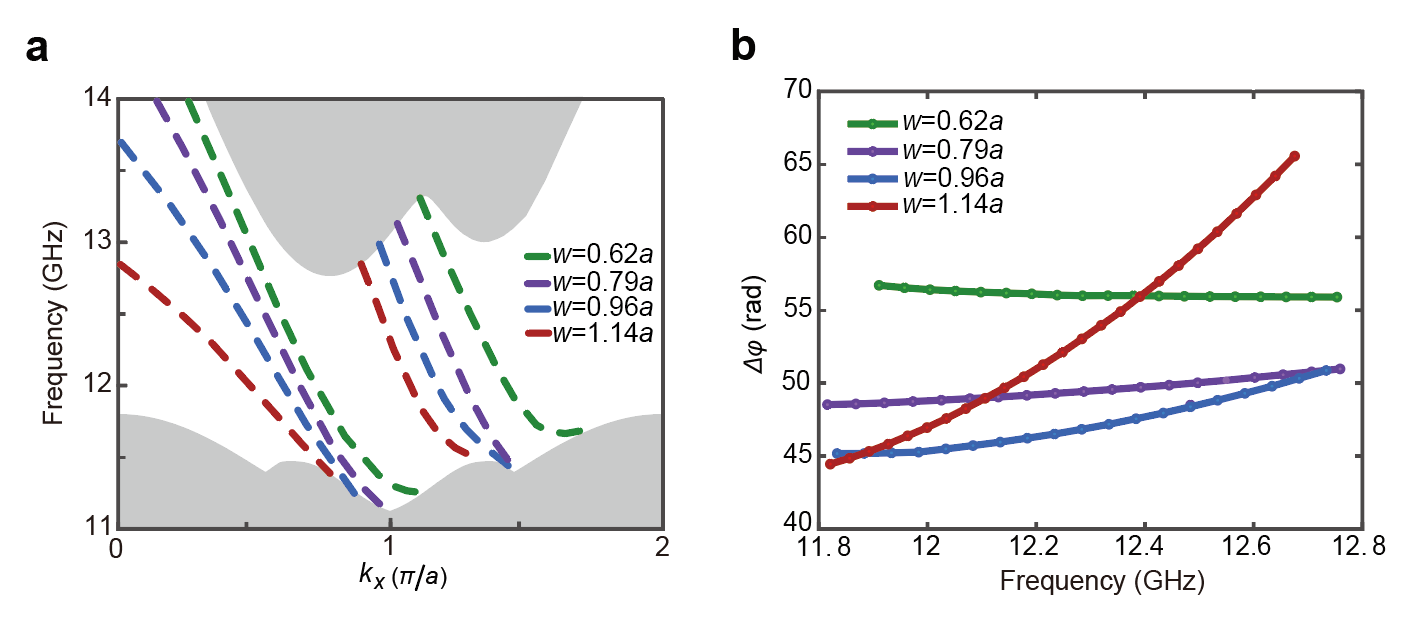


**Fig. S3 The effect of the width of air gap on the dispersions of edge modes and their phase differences. a** The dispersions of edge modes vary with different size of air gap. **b** The phase differences of two CEMs as functions of frequency with air gaps of different width.


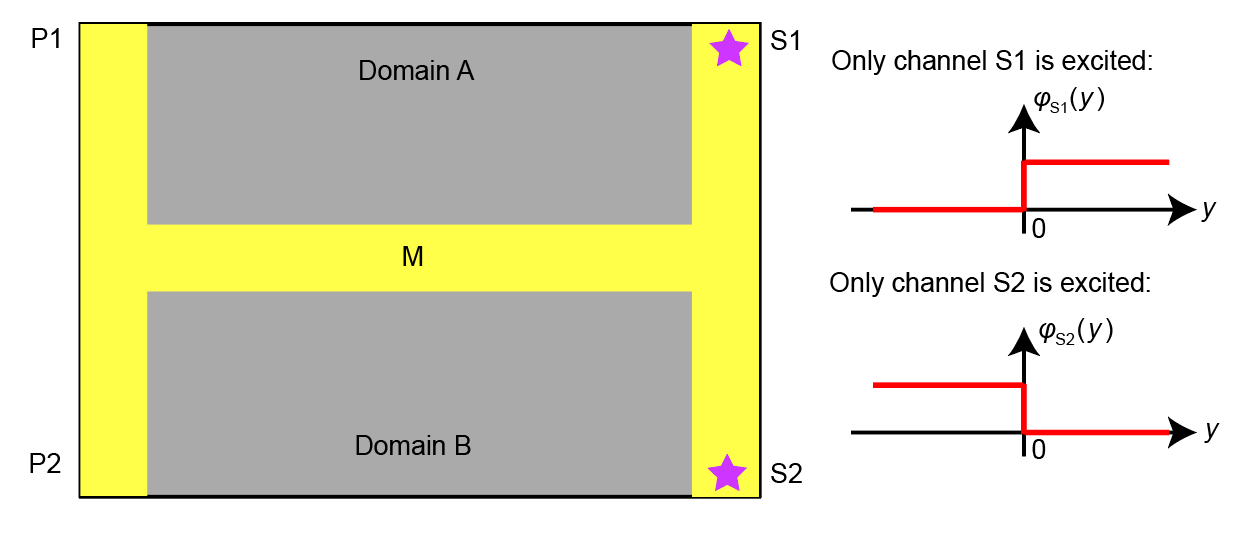


**Fig. S4** **The sketch of right-hand-side intersection and the incident signal along y direction.** The stars indicate the positions of sources.
